# Beauty Choices: Uncovering the Profile of Who Opts for Aesthetic Procedures

**DOI:** 10.1007/s00266-025-05011-7

**Published:** 2025-06-19

**Authors:** Rafaela Rocha Agrizzi, Matheus Bellanda Peroni, Leticia Machado Gonçalves-Soares, Ana Claudia Carbone, Mariana Barbosa Câmara-Souza, Alfonso Sanchez-Ayala, Ana Cristina Manso, Giancarlo De la Torre Canales

**Affiliations:** 1 Egas Moniz Center for Interdisciplinary Research (CiiEM), Egas Moniz School of Health and Science, Caparica, Almada Portugal; 2 Department of Dentistry, Federal University of Maranhao, Sao Luis, Maranhao Brazil; 3 Department of Dentistry, Ingá University Center, Uningá, Paraná Brazil; 4 Department of Dentistry, University of Ponta Grossa, Ponta Grossa, Paraná Brazil; 5 Division of Oral Rehabilitation, Department of Dental Medicine, Karolinska Institutet, Huddinge, Sweden

**Keywords:** Aesthetic procedures, Women, Sociodemographic factors

## Abstract

**Background:**

The demand for aesthetic procedures has increased, driven by advances in aesthetic therapies. Therefore, understanding the factors that influence individuals to undergo aesthetic procedures is important. This study aimed to assess the profile of patients that received or did not receive aesthetic procedures.

**Methods:**

This cross-sectional study enrolled 834 female participants aged between 18 and 65 years which were divided in two groups: yes to aesthetic procedures (YAP) and no to aesthetic procedures (NAP). Participants were instructed to fill out a form that included sociodemographic questions, a question regarding performing or not aesthetic procedures and the Portuguese validated versions of five FACE-Q scales. Data were collected electronically from October 2023 to February 2025. For group comparison for continuous variables, the Yuen test was used and Fisher's exact test for categorical variables. Multivariate logistic regression was also performed to assess the influence of variables in performing aesthetic procedures.

**Results:**

The sociodemographic data showed group differences in age, education and marital status and income (*p* < 0.001). Botulinum toxin type A was the most common minimally invasive procedures reported by the participants. Also, volunteers of the YAP group showed greater self-perception and concern about ageing (FACE-Q1 ageing appraisal) (*p*=0.001). Multivariate logistic regression analysis showed that marital and education status, income and FACE-Q1 influence the decision of performing aesthetic procedures. *Conclusion:* Women of higher educational level, divorce, with higher income and that present higher concerns about ageing are linked to a greater likelihood of undergoing facial aesthetic procedures.

**Level of Evidence III:**

This journal requires that authors assign a level of evidence to each article. For a full description of these Evidence-Based Medicine ratings, please refer to the Table of Contents or the online Instructions to Authors  www.springer.com/00266.

## Introduction

The perception of beauty has been a topic of debate throughout history, primarily due to its inherent complexity and subjectivity [1]. The increasing pursuit of aesthetic standards, largely influenced by social media, underscores the impact of appearance on self-esteem and quality of life. Facial ageing, being particularly conspicuous, has become a primary focus of aesthetic interventions aimed at mitigating the signs of ageing [1]. Associated changes with facial ageing, including dynamic wrinkles, skin laxity and bone resorption, are influenced by both genetic and environmental factors [2, 3]. A comprehensive understanding of these processes is essential for evaluating the impact and effectiveness of aesthetic interventions.

In this context, the demand for aesthetic procedures has increased, driven by advances in both surgical and non-surgical therapies [4]. According to data from the International Society of Aesthetic Plastic Surgery, the volume of aesthetic and cosmetic procedures performed in 2023 experienced a global increase of 80.1% compared to the past four years (2019), comprising 15.8 million surgical and 19.2 million non-surgical interventions [5]. The USA leads in the number of non-surgical procedures performed (4.4 million), while Brazil ranks highest for surgical procedures (2.18 million) [5]. Notably, botulinum toxin injections emerged as the most common cosmetic procedure across all age groups, accounting for 46.4% of total non-surgical interventions. This growing demand reflects an increasing interest in minimally invasive techniques to counteract signs of ageing.

While many patients report enhanced self-esteem and well-being following these interventions, studies have raised psychosocial concerns associated with aesthetic procedures [6]. Significant gaps persist in understanding the psychosocial factors that drive this demand and in assessing the long-term emotional impacts of such interventions [7]. A cross-sectional observational study revealed that individuals seeking aesthetic procedures exhibited higher levels of anxiety, depression and interpersonal sensitivity compared to those who did not pursue such treatments [7]. Furthermore, Wang et al. [8] identified increased anxiety disorders following facial aesthetic interventions, a phenomenon referred to as post-injection cosmetic emotional distress syndrome. Conversely, the literature suggests that individuals often perceive themselves as more attractive than they truly are [9]. This observation raises the hypothesis that the pursuit of aesthetic procedures may be associated with underlying social and psychological deficits, and in some instances, with body dysmorphic disorder, which may not be adequately addressed by any aesthetic intervention [10].

Therefore, understanding the motivations and perceptions of individuals who seek or have undergone aesthetic procedures is crucial for ensuring responsible interventions and minimizing adverse psychological effects. Additionally, knowledge of the sociodemographic and psychosocial characteristics of these patients can enhance the aesthetic market and inform clinicians'approaches to patient care. Therefore, this study aims to characterize the profile of individuals who have received aesthetic procedures compared to those who have not, as well as to identify the determinants influencing these decisions.

## Material and Methods

This multicentre cross-sectional online survey study was approved by the Research Ethics Committee of  Egas Moniz School of Health & Science, Portugal (PT-210/24) and Ingá University Center, Uningá, Brazil (CAAE: 73724923.0.0000.5220). All participants provided a signed consent informing that they would like to participate in the study. The reporting of the data followed the Strengthening the Reporting of Observational Studies in Epidemiology (STROBE) guideline.

### Participants

The sample was obtained from Portuguese and Brazilian individuals. Inclusion criteria were women, aged between 18 and 65 years, that performed or not aesthetic procedures in the face region and with complete dentition or using functional and adapted dental prosthodontics, since tooth lose can generate facial aesthetic alterations. Exclusion criteria were patients with craniofacial deformities and facial skin burn injuries, because these facial alterations are not related to ageing. Also, people with diagnosed psychosocial disorders were not included, since they could have an alter perception of themselves.

The sample size calculation was based on a previous study [7] assessing the psychological profile of women seeking for aesthetic procedures, with the G*Power 3.1.9.2 software (Kiel, Germany). The following parameters were considered: a power of 0.95, a significance level of 0.05 and an effect size of 0.5. The actual power obtained with this sample size was 95.23%. The final sample was composed of 824 participants, divided into two groups: 416 in the group yes to aesthetic procedures (YAP) and 408 in the group that no to aesthetic procedures (NAP). Participants were assigned to the groups based on the question: Have you ever undergone any facial aesthetic procedure? If, so, which one(s)?

### Study Protocol

Participants were assessed once in this study. For this, a form was created in Google Forms which consisted in three parts a) inform consent, b) sociodemographic questions and a question regarding aesthetic procedures and c) Portuguese validated version of five FACE-Q scales. The study advertisement was performed via websites and social media and included a link to the proposed Google forms. Data were collected electronically from October 2023 to February 2025.

### Outcomes

#### Sociodemographic Questionnaire

The collected sociodemographic data included questions regarding country of birth (Brazil or Portugal), age (≤ 18, 19–30, 31–45, 46–60 and > 60), marital status (single, married, divorce and stable union), educational level (incomplete high school, high school, undergraduate and postgraduate) and income (very low, low, lower middle, upper middle and high). Regarding income data, the categories were matched accordingly to each country income.

#### FACE-Q Scales

The FACE-Q scales are validated instruments designed to measure outcome expectations and individuals’ satisfaction prior to undergoing facial treatments and after treatments [11, 12]. In this study, the following FACE-Q appraisal assessed health-related quality of life: *FACE-Q1 ageing appraisal, FACE-Q2 psychological function, FACE-Q3 appearance-related psychosocial distress and FACE-Q5 social function,* using a 4-point scale where 1 indicates"totally disagree", 2 represents"somewhat disagree", 3 signifies"somewhat agree"and 4 denotes"totally agree"[13]. Also, the *FACE-Q4 satisfaction with facial appearance* which evaluates the extent to which participants were satisfied with their face appearance, using a 4-point scale where 1 signifies"very dissatisfied", 2 indicates"somewhat dissatisfied", 3 represents"somewhat satisfied"and 4 denotes"very satisfied"was used. Scoring of all these scales involve summing the individual item scores to generate a total raw score, which is then converted, using a specific conversion table, into a range from 0 (indicating the worst outcome) to 100 (representing the best outcome) [14].

The authors of this study have secured a licence agreement to employ the FACE-Q scales for non-profit academic research purposes.

### Data Analysis

Data from the survey were explored using R (version 4.4.3, R Foundation for Statistical Computing, Vienna, Austria). A *p*-value of <0.05 was considered significant. The variables were initially analysed descriptively, using the median for quantitative measures, as well as frequency estimates for qualitative variables.

Principal component analysis with mixed data (PCAmix) was conducted to reduce the dimensionality of the data and assess the performance of the predictive model. Afterwards, a logistic regression analysis was conducted. The number of components retained in PCAmix was determined based on eigenvalues greater than 1. Logistic regression analysis utilized principal components as predictors for the aesthetic procedures’ variable. The absence of multicollinearity (a high correlation between independent variables) was assessed using the variance inflation factor (VIF). Additionally, the Hosmer–Lemeshow test was performed to evaluate the model's fit, or how well the developed model corresponds with the data. To assess the model's performance in terms of sensitivity and specificity, odds ratios and their confidence intervals were also calculated. The correlation matrix for the numerical variables, along with the graphs for sedimentation, variables from the multiple correspondence factor analysis (MCA) and the arrangement of categorical variables in the PCA space, was also prepared for improved visualization during analysis.

The comparison of groups for continuous variables was conducted using the Yuen test (with a trim proportion of 20% to mitigate the influence of extreme values) and Fisher's exact test for categorical variables.

## Results

### Sociodemographic Characteristics

The sociodemographic characteristics of the 834 women who participated in the study are summarized in Table 1. It was observed that the age range of 31–45 years was more frequent in the YAP group, whereas the age range of 19–30 years was more common in the NAP group. In both groups, most women were single. Regarding educational level and income, 66% of the YAP group had a postgraduate degree, and 29% had an upper middle income. In contrast, the NAP group consisted mostly of undergraduate individuals (35%) and those with very low income (47%). There were statistically significant differences between the groups for all sociodemographic variables (*p* < 0.001). Minimally invasive procedures were the most reported by the participants, with botulinum toxin type A been the most common one (35%), followed by skin treatments (29%) and hyaluronic acid fillers (25%). Invasive procedure represented 10% of the aesthetic procedures.

**Table 1:** Frequencies % (*n*) of demographic characteristics of the study population

	Groups		
	YAP (*n* = 416)	NAP (*n* = 408)	*p*
*Country*			
Portugal	17 (71)	50 (206)	0.001^*^
Brazil	83 (345)	50 (202)	
*Age*			
≤ 18	2 (7)	5 (22)	0.001^*^
19–30	33 (136)	67 (272)	
31–45	40 (165)	20 (80)	
46–60	23 (96)	8 (33)	
> 60	3 (12)	0 (1)	
*Marital status*			
Single	39 (161)	67 (272)	0.001^*^
Married	38 (160)	20 (82)	
Divorce	12 (48)	3 (14)	
Stable union	11 (47)	10 (40)	
*Education*			
Incomplete high school	1 (5)	4 (18)	
High school	10(43)	34 (137)	
Undergraduate	23 (94)	35 (141)	0.001^*^
Postgraduate	66 (274)	27 (112)	
*Income*			
Low	14 (57)	47 (192)	
Lower middle	23 (94)	34 (139)	
Middle	29 (121)	12 (50)	0.001^*^
Upper middle	24 (99)	6 (23)	
High	11 (45)	1 (24)	

Income: Both countries were matched in the present classification accordingly to their income status, independent of their currency

*YAP* yes to aesthetic procedures, *NAP* no to aesthetic procedures

*p<0.05 between groups

#### FACE-Q Appraisals

Inter-group comparisons showed statistically significant differences in ageing appraisal and appearance-related psychosocial distress (Table 2). Individuals in the YAP group presented greater self-perception and concern about ageing (FACE-Q1 ageing appraisal) (*p* = 0.001), whereas individuals of the NAP group reported greater psychosocial distress regarding their appearance (*p* = 0.01). No impact was observed in psychological function, satisfaction with appearance and social function between the assessed groups (*p* > 0.05).

**Table 2:** Median (min–max) of FACE-Q questionnaires scores according to groups

	Groups		
	YAP (*n* = 416)	NAP (*n* = 408)	*P*
FACE-Q1	77 (0–100)	46 (0–100)	0.001*
FACE-Q2	31 (0–100)	26 (0–100)	0.57
FACE-Q3	51 (0–100)	55 (0–100)	0.01*
FACE-Q4	71 (0–100)	74 (0–100)	0.31
FACE-Q5	58 (0–100)	58 (0–100)	0.35

*YAP* yes to aesthetic procedures, *NAP* no to aesthetic procedures

*p<0.05 between groups

#### Predictors for Doing Aesthetic Treatments

**Fig. 1:**
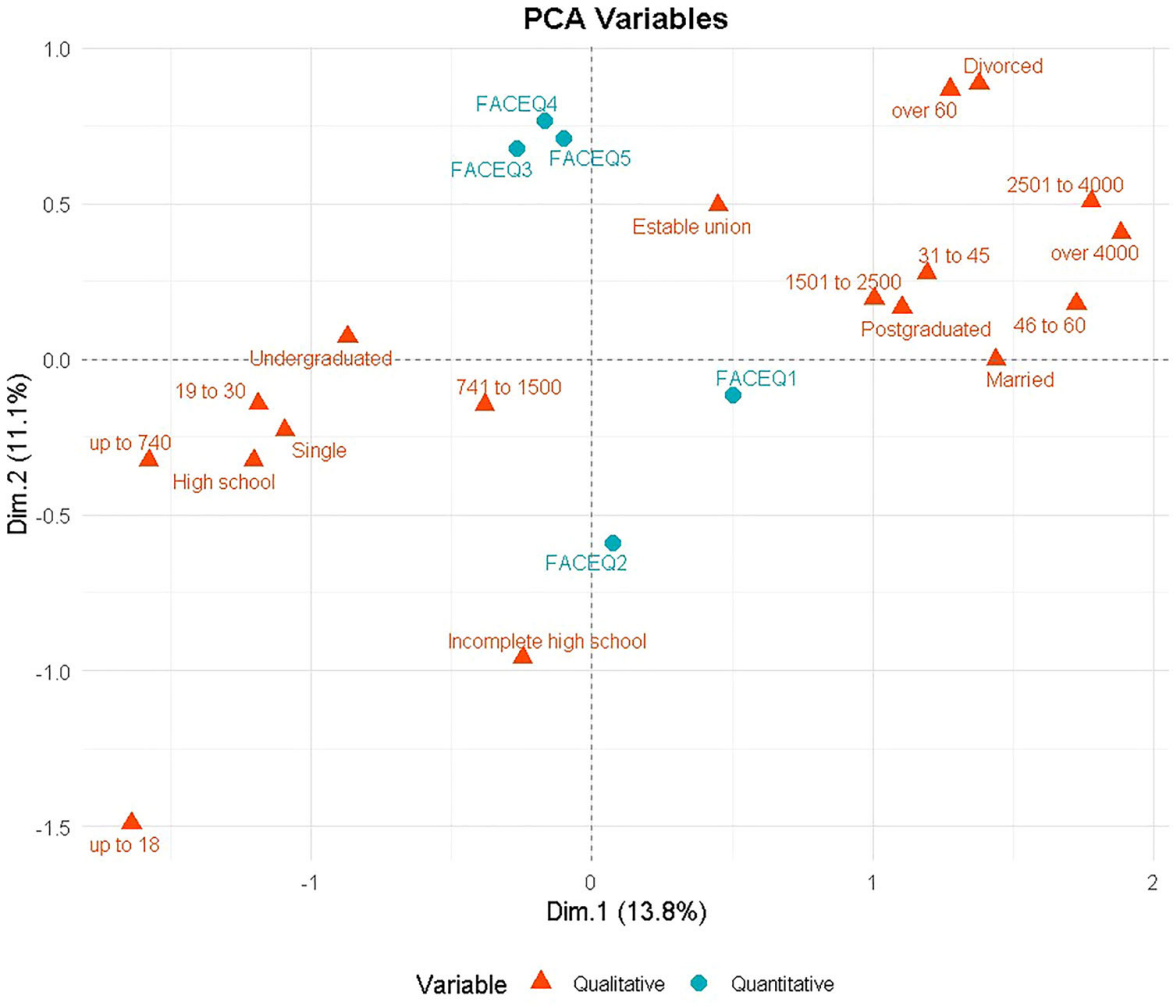
Principal component analysis (PCAmix) of volunteers that performed or not aesthetic procedures. Loading plot showing the variables and their loadings. Boxes in the upper right direction are significant variables. Orange triangles are qualitative variables, and blue circles are quantitative variables

Figure 1 shows the distribution of qualitative variables, represented by orange triangles (age, marital status, education level and income); and quantitative variables, represented by blue circles (FACE-Q1, FACE-Q2, FACE-Q3, FACE-Q4 and FACE-Q5). There appears to be a clear segmentation based on income (horizontal), and marital status and education level (vertical). For example, perception of ageing (FACE-Q1) seems to be more associated with higher income and education level, whereas greater psychosocial distress regarding appearance (FACE-Q2) shows a positive correlation with younger profiles or different marital status.

A previous linear logistic regression was performed using sociodemographic data and FACE-Q questionnaires as predictors for the aesthetic procedure variable. Among the main components analysed (5), only the interaction of age, income, marital status, educational level and ageing appraisal (FACE-Q1) was statistically significant (*p* < 0.0001). Also, it has an OR of 0.455 (CI 0.405–0.508), indicating that each increase in these components reduces the likelihood of the event occurring by approximately 54.5%. Since only this interaction showed statistical significance, the probability of undergoing aesthetic procedures was modelled based on the predictor variables: age, marital status, education level, income and FACE-Q1 (Table 3).

**Table 3:** Multivariate logistic regression coefficients of principal predictor variables for the probability of undergoing aesthetic procedures (YAP group)

	Estimate	SE	*Z* value	*p*
Intercept	−2.54	0.46	−5.49	0.00001^*^
*Age*				
≤ 18	−0.44	0.66	−0.66	0.50
19–30	−0.24	0.29	−0.82	0.41
*31–45*				
46–60	0.47	0.37	1.26	0.20
> 60	1.50	1.37	1.09	0.27
*Marital status*				
Single	−0.16	0.29	−0.56	0.57
*Married*				
Divorce	1.09	0.51	2.13	0.03^*^
Stable union	0.06	0.38	0.16	0.866
*Education*				
Incomplete high school	−1.25	1.27	−0.98	0.32
*High School*				
Undergraduate	0.76	0.29	2.58	0.009^*^
Postgraduate	1.57	0.30	5.24	0.0001^*^
*Income*				
*740*				
741–1500	0.51	0.27	1.85	0.06
1501–2500	0.99	0.33	2.93	0.003^*^
2501–4000	1.40	0.42	3.31	0.0009^*^
4000	2.25	0.71	3.13	0.001^*^
*FACE-Q*				
FACE-Q1	0.01	0.00	5.26	0.00001^*^

Income was matched according to each country income category

FACE-Q1: ageing appraisal

*p<0.05

Multivariate logistic regression showed that age variable did not show statistically significant effects (*p* > 0.05), suggesting that age alone does not have a relevant impact on the probability of undergoing aesthetic procedures. Marital status was significant only for the “divorced” category (*β* = 1.093, *p* = 0.032), indicating that divorced individuals are more likely to undergo aesthetic procedures compared to with the other marital status category. Education level significantly influenced the decision to undergo procedures. Undergraduate individuals (*β* = 0.769, *p* = 0.0098) and, more notably, those with a postgraduate degree (*β* = 1.577, *p* < 0.001) had a significantly higher probability of undergoing aesthetic procedures compared to those with lower educational levels. Income also had a significant impact. All income above $1,500 (middle) had positive and statistically significant coefficients, with the effect increasing as income rises. Individuals in the upper middle and high income had substantially higher chances of undergoing aesthetic procedures. Finally, the FACE-Q1 variable (*β* = 0.017, *p* < 0.001) also had a significant positive effect, suggesting that an increased perception of ageing is associated with a higher probability of undergoing aesthetic procedures.

## Discussion

With the increasing demand for facial cosmetic procedures, it is essential to understand the characteristics of patients seeking or undergoing these treatments to effectively deliver patient-centred care. This study presents evidence regarding the motivations of individuals who pursue or have received aesthetic procedures, while also investigating the impact of sociodemographic factors on their decision-making processes. Our findings indicate that the profile of patients undergoing aesthetic procedures is significantly influenced by variables such as marital status, educational attainment, income level and perceptions of ageing.

The present study utilized the FACE-Q Appraisals instrument to assess the quality of life and perceptions of ageing among volunteers. The FACE-Q effectively measures patient satisfaction across various domains, including overall facial appearance, specific facial regions, psychological well-being, age appraisal and adverse effects [15]. Our findings indicate that the primary motivation for patients seeking aesthetic procedures is their concern regarding ageing, particularly facial ageing, which can significantly affect self-esteem and psychological well-being [16]. Notably, previous research has similarly identified concerns about ageing among patients utilizing neuromodulators and facial fillers [17–19]. Furthermore, these studies have demonstrated that combined treatment approaches generally enhance age appraisal more effectively than single procedures alone. Consequently, to optimize patient satisfaction and retention, practitioners should consider implementing a combined treatment strategy that includes both fillers and neuromodulators rather than relying on either modality in isolation. This approach has the potential to substantially improve patients'quality of life [17–20]. Our study revealed a low percentage of participants who had received combined procedures, which may explain the elevated scores in the FACE-Q ageing appraisal. Further research is warranted to compare various combined treatment approaches, as this could yield more robust recommendations regarding the effects of these interventions on satisfaction with appearance and perceptions of ageing [15]. In contrast, our findings from the FACE-Q module on psychosocial distress concerning appearance [14] indicate that patients who did not undergo aesthetic procedures reported a greater emotional and social impact related to their appearance. It is plausible that individuals may experience some form of distress or psychological need, particularly in social contexts, regarding their appearance. As a result, they might view these procedures as potential solutions for addressing perceived deficiencies that arise from socially established beauty standards, despite personally believing they do not require such interventions. Understanding the psychosocial impact of aesthetic treatment provides valuable insight into the motivations driving individuals to initially seek these interventions and enables clinicians to inform patients about the significant emotional effects associated with such procedures [15].

Interestingly, no significant differences were observed among the groups concerning psychological functioning, overall satisfaction with appearance, or social functioning. This finding suggests that undergoing an aesthetic procedure alone may be insufficient for patients to experience substantial psychological and social benefits, particularly given the increasingly demanding and varied contemporary aesthetic standards. However, it is important to note that the lack of standardization regarding the timing of procedures within the sample may account for these findings. Previous research has indicated that psychological function scores can change over time, typically peaking one month after the administration of neuromodulators or fillers, followed by a gradual decline thereafter [19, 21]. Thus, the psychological advantages may be immediate or persist only as long as the treatment remains effective.

Regarding the sociodemographic profile of the sample, our study found that age alone does not significantly influence the likelihood of undergoing aesthetic procedures. A previous study utilizing the FACE-Q expectations scale indicated that individuals aged 30–39 years exhibited higher expectations regarding aesthetic procedures compared to older age groups. This suggests a potential desire among this demographic to proactively address perceived changes associated with ageing [22]. However, this heightened expectation does not necessarily correlate with a higher likelihood of undergoing aesthetic procedures. In contrast, our study found that age was not a determinant factor for pursuing aesthetic interventions. This discrepancy may be attributed to differences in sample size and the focus of our study specifically on patients who had already undergone aesthetic procedures. It is also plausible that, regardless of age, there exists a notable concern about the ageing process, distorted self-perception of aesthetics and varying degrees of social impairment. Our findings concerning age also differ from those of another study that reported that participants aged 30–45 years were more likely to seek aesthetic treatments; however, that study included participants of both sexes and focused specifically on undergoing minimally invasive aesthetic procedures [23].

When examining marital status, it was observed that divorced individuals were more likely to undergo aesthetic procedures compared to those in other marital categories. The dissolution of a marriage is associated with elevated levels of depressive symptoms and decreased life satisfaction [24, 25]. It is plausible to suggest that divorced women may perceive undergoing aesthetic procedures as a means to enhance mental health, self-esteem, attractiveness and confidence in seeking new relationships. With respect to educational attainment, individuals with higher education credentials (including undergraduate and postgraduate degrees) exhibited a significantly greater likelihood of pursuing aesthetic procedures compared to individuals with lower educational levels. It is well established that individuals with advanced education tend to secure higher-paying employment, thereby affording the often-substantial costs associated with aesthetic procedures. Furthermore, evidence indicates that physical attractiveness may increase the likelihood of being selected for prestigious PhD programmes among women [26]. Additionally, individuals within this educational cohort acknowledge that while physical appearance is not the sole determinant, it can significantly influence professional success, career opportunities and earning potential [27]. Therefore, the findings from our study suggest that marital status and educational attainment are influential factors in the decision to undergo aesthetic procedures, in contrast to other studies that reported that relationship status, educational attainment and even employment status were not significant predictors of pursuing aesthetic treatments [23].

As anticipated, income also had a significant impact on the results, consistent with previous findings [23]. Since these procedures are elective and require financial investment, individuals with upper middle and high incomes are substantially more likely to pursue aesthetic treatments than those with lower incomes. Furthermore, financial independence has been identified as a key predictor of a more positive self-perception of ageing. Participants who reported high levels of financial stress were more likely to perceive themselves as older than their actual age and experienced a greater increase in perceived age over time [28], which may drive them to seek multiple aesthetic procedures.

This study provides important insights into the sociodemographic and psychosocial profiles of individuals who opt for aesthetic procedures. Our findings can inform the aesthetic industry about which demographic cohorts may be the primary focus for marketing and sales and enhance healthcare professionals'approaches to patient care by elucidating the characteristics of the patients they will encounter in their clinics. During patient assessments, clinicians can use these insights to better identify individuals who may have higher expectations or emotional investments in treatment outcomes. For example, patients with greater ageing appraisal scores (FACE-Q1) may benefit from comprehensive education about realistic results and timelines, or even psychological support when appropriate. Furthermore, awareness of these profiles allows practitioners to adopt a more individualized and empathetic approach, enhancing patient trust and satisfaction. By recognizing the potential psychosocial drivers behind aesthetic procedure requests, clinicians can engage in more meaningful dialogue, set appropriate expectations and ultimately contribute to safer and more ethical aesthetic care. However, there are several limitations to consider. Firstly, the study exclusively included female participants, which may restrict the generalizability of the findings to other populations. Additionally, the inclusion of only Portuguese and Brazilian females may limit the applicability of the results to other cultural or ethnic groups. Furthermore, data regarding the time elapsed since the aesthetic procedures were performed were not collected for the cohort undergoing these interventions, thereby limiting certain inferences. It is also important to interpret the results with caution, as self-reported surveys are subjective assessments reliant solely on patient reports. Finally, future studies should assess the impact of aesthetic procedures on the psychosocial status of various populations over time in a broader context.

## Conclusion

In conclusion, the decision to undergo aesthetic procedures among women is primarily influenced by sociodemographic factors and individual perceptions of ageing. Specifically, women—irrespective of age—who possess higher levels of educational attainment, are divorced, and have higher incomes represent the demographic most likely to seek aesthetic interventions.
